# Co-expression of neighbouring genes in Arabidopsis: separating chromatin effects from direct interactions

**DOI:** 10.1186/1471-2164-11-178

**Published:** 2010-03-16

**Authors:** Wei-Hua Chen, Juliette de Meaux, Martin J Lercher

**Affiliations:** 1 Bioinformatics, Heinrich-Heine University Duesseldorf, 40225, Germany; 2 European Molecular Biology Laboratory (EMBL), Meyerhofstrasse 1, 69117 Heidelberg, Germany; 3 Max Planck Institute for Plant Breeding Research, Carl-von-Linné Weg 10, 50829 Cologne, Germany

## Abstract

**Background:**

In all eukaryotic species examined, genes that are chromosomal neighbours are more similar in their expression than random gene pairs. Currently, it is still unclear how much of this local co-expression is caused by direct transcriptional interactions, and how much is due to shared chromatin environments.

**Results:**

We analysed neighbouring genes in *Arabidopsis thaliana*. At large intergenic distances (>400 bp), divergently and convergently transcribed gene pairs show very similar levels of co-expression, mediated most likely by shared chromatin environments. At gene distances below 400 bp, co-expression is strongly enhanced only for divergently transcribed gene pairs, indicating bi-directional transcription from a single promoter. Conversely, co-expression is suppressed for short convergently or uni-directionally transcribed pairs. This suppression points to transcriptional interference concentrated at the 3' end, e.g., in the context of transcription termination.

**Conclusions:**

Classifying linked gene pairs by their orientation, we are able to partially tease apart the different levels of regional expression modulation. (i) Regional chromatin characteristics modulate the accessibility for regulation and transcription, regardless of gene orientation; the strength of this chromatin effect can be assessed from divergently or convergently transcribed distant neighbours. (ii) Shared promoter regions up to 400 bp in length enhance the co-expression of close bi-directional neighbours. (iii) Transcriptional interference of close neighbours is concentrated at the 3' ends of genes, and reduces co-expression on average by 40%.

## Background

Eukaryotic gene order is not random with respect to gene functions or expression patterns: clusters of co-expressed genes are routinely observed in a wide range of species [1]. For example, 'housekeeping' genes expressed in many tissues tend to cluster in humans [2]; directly neighbouring gene pairs are more likely to act in the metabolic pathway [3]; and neighbouring genes that are transcribed divergently (←→) are unusually frequent in the human genome and show particularly strong co-expression [4]. Co-expression is not restricted to close neighbours; in yeast, significant co-expression is observed over distances covering dozens of genes [5].

Several mechanisms have been proposed to explain this local co-expression of neighbouring gene pairs, including shared promoters or transcription factor binding sites, transcriptional read-through, and chromatin remodelling [1,6]. In the yeast genome, for example, much local co-expression may stem from chromatin domains that as a whole switch between euchromatin and heterochromatin states [7]. It is not clear to what extent this observation can be extrapolated to other model species: in mammalian genomes, there appears to be no corresponding compartmentalization of tissue-specific genes into co-regulated regions [6]. Thus, the relative contributions of different local co-expression mechanisms seem to vary across species [1]. This may not be surprising: fine details of gene regulation are under strong, species-specific selective pressures, and are affected by multiple internal and external cellular factors [8,9].

Due to the compactness of its genome [10], the thale cress *Arabidopsis thaliana *is a multi-cellular eukaryote particularly well suited to the study of local co-expression. Short intergenic regions mean that neighbouring genes are physically very close to each other. As all proposed mechanisms for local co-expression are likely to act in a distance-dependent way [1], this should enhance their signal compared to genomes with larger and more variable gene distances. Several previous studies have examined local co-expression in *A. thaliana*.

Similar to previous results in fungi and animals [reviewed in [1]], neighbouring genes in *A. thaliana *are on average more co-expressed than expected by chance [11]. 5-10% of *A. thaliana *genes are located in highly co-expressed clusters of 2-4 neighbouring genes [12,13]; however, these are mostly not in the same Gene Ontology (GO) categories, and there is no detectable micro-syntenty of co-expressed clusters to corresponding clusters in rice [14]. Evolutionary conservation of gene neighbourhood was stronger for highly co-expressed gene pairs, as well as for gene pairs sharing at least one GO category. The genes in 60% of metabolic pathways show significant clustering on *A. thaliana *chromosomes, a feature also found in other eukaryotes [3,11]. While co-expresion of tandemly duplicated genes provides some of the signal, neighbour co-expression remains significant even when excluding duplicated genes [3,11,13].

Several previous studies have examined the role of relative gene orientation in determining co-expression levels of neighbouring genes, and have found that divergent transcription can account for some but not all of the co-expression effect [11-13,15]. Divergent transcription of *A. thaliana *is preferentially associated with certain GO categories [16], and co-expression is increased between genes of related functions [15]. Thus, while the possible sharing of promoter regions explains part of the co-expression pattern, it is highly likely that chromatin effects also play an important role in *A. thaliana*. This is supported by the finding that duplicated genes which are parts of larger duplications retain strongly correlated expression patterns, as long as their neighbourhood is conserved [17].

In this study, we dissect co-expression patterns of neighbouring gene pairs in the model plant species *A. thaliana*, paying particular attention to the relative orientation of the genes and to intergenic distances. We find the strongest co-expression among uni-directional gene pairs; this contrasts with results in humans and yeast, where bi-directional gene pairs are the most strongly co-expressed. This contradiction is resolved when examining only closely spaced gene pairs (<400 bp), where bi-directional pairs are the most strongly co-expressed also in *A. thaliana*. In line with expectations, this indicates that sharing of regulatory elements enhances co-expression. The opposite effect is seen for shared 3' regions in convergent gene pairs: these show decreased co-expression levels. The latter observation appears consistent with a model of transcription termination in which the polymerase overshoots the polyadenylation addition site [18-20], and may hence interfere with the transcription of closely positioned downstream genes.

## Results and discussion

### Co-expression extends over large distances

**Figure 1:**
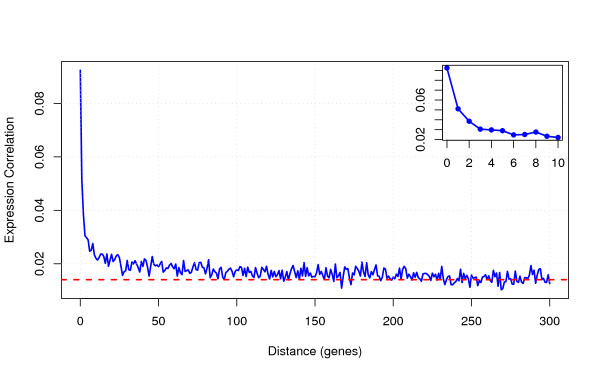
**Co-expression decreases with increasing distance (Inset: blow-up of 0-10 intervening genes)**. Co-expression is strongest for direct neighbours (distance = 0), drops quickly up to 2 intervening genes, and then slowly decreases until it reaches average values for non-neighbours (dashed line) at very large distances. Co-expression of direct neighbours is significantly higher than for random pairs regardless of gene orientations (Table 1), and co-expression is higher than for random pairs up to 100 intervening genes (P < 0.02 for each distance bin). Co-expression is measured as Pearson's correlation coefficient of gene expression vectors across experiments. Distance is measured as the number of intervening genes along the chromosome.

Using expression data across 1,436 microarray experiments (see methods), we calculated the Pearson correlation coefficients for all pairs of neighbouring protein coding genes. The co-expression values range from -1 to 1, with values close to 1 indicating high co-expression, and values close to -1 indicating strong anti-correlation of expression. Co-expression values were averaged over all pairs with the same distance (number of intervening genes) [NB: averaging over signed correlation coefficients does not allow to distinguish lack of co-expression from a superposition of equal amounts of positive and negative co-expression; however, this is justified as the overall distribution of co-expression values is approximately Gaussian]. As shown in Figure 1, genes in close proximity tend to be highly co-expressed. This co-expression extends at least 100 genes along the genome: mean co-expression of all gene pairs with the same number *d *of intervening genes is significantly higher than for random pairs (*P *< 0.02 for all *d *< 100, Wilcoxon rank sum tests). Thus, co-expression clusters seem to extend further than previously considered [11]. Co-expression rapidly drops as soon as there are any intervening genes (see inset of Figure 1). This is consistent with previous reports on Arabidopsis, which found that strongly co-expressed clusters are small (2-4 genes) [12,13].

Why does co-expression drop so fast? Consider three consecutive genes A-B-C. If both A-B and B-C are correlated in their expression, shouldn't it follow that A-C are correlated similarly? Assume that the corresponding expression patterns (a, b, c) are correlated such that variation in *a *explains a fraction *R*_*ab*_^2 ^of variation in *b*, and variation in *b *explains a fraction *R*_*bc*_^2 ^of variation in *c*. If there is no direct interaction of *a *and *c*, we would expect a correlation *R*_*ac*_^2 ^= *R*_*ab*_^2 ^× *R*_*bc*_^2 ^between *a *and *c*. If we insert the numbers read from Figure 1, we thus expect a co-expression value of approx. 0.01 for pairs with 1 intervening gene, which is actually substantially lower than the observed value. This indicates that there are indeed local factors that enhance co-expression for pairs that are not direct neighbours.

### Different orientations show different co-expression levels

By far the strongest co-expression is seen for direct neighbours, on which we focused next. To ensure *a priori *independence of the genes, we removed all genes that overlap with other (protein coding or non-coding) genes in the genome according to the TAIR7 annotation [21,22]. The remaining 24,638 pairs of non-overlapping protein coding gene pairs were further classified according to their relative orientations, resulting in 6,077 bi-directional (or divergent, ←→), 13,574 uni-directional (→→ or ←←), and 4,987 convergent pairs (→←).

For all three types, average co-expression of neighbouring gene pairs was significantly stronger compared to random pairs (Table 1), consistent with previous results [11,12,15]. While average co-expression is positive, we found that 42.8% of neighbouring pairs show anti-correlated expression (compared to 50.4% in random pairs); similar distributions were observed in humans [4].

**Table 1:** Co-expression of directly neighbouring genes

Type	Co-expression *	P** (compared to random pairs)
Bi-directional	0.084	<10^-15^
Uni-directional	0.088	<10^-15^
Convergent	0.035	0.0067

* Average Pearson correlation coefficient

** To derive a Null expectation, we analyzed 100,000 pairs of randomly chosen genes; mean Pearson correlation of this random dataset is 0.014. *P*-values from Wilcoxon rank sum tests.

Among the three relative orientations, uni-directional pairs showed the highest average expression correlation, as was also observed previously [13]. This contrasts with findings in the human genome, where bi-directional pairs are more highly co-expressed [4]. Due to the vast evolutionary distance and different ecology, direct comparisons are of course difficult; however, one possible reason for this difference between humans and plants is the existence of bicistronic and fused monocistronic transcripts in the *A. thaliana *genome [23]. Bicistronic transcripts contain two separate open reading frames (ORFs), leading to perfect co-expression of the ORFs; fused monocistronic transcripts arise by the fusion of two adjacent transcripts in particular tissues or environments, leading to elevated co-expression. To test if the higher average co-expression of uni-directional pairs might be attributed to bicistronic and fused monocistronic transcripts, we used a previously determined set of such genes (58 bicistronic and 30 fused monocistronic transcripts [23]). We found an average co-expression of 0.108, only slightly higher than the value obtained for all uni-directional transcripts. After excluding these bicistronic and fused monocistronic transcripts, uni-directional gene pairs are still more co-expressed than the two alternative relative orientations (*P *= 3.2 × 10^-10 ^and 0.016 for convergent and bi-directional pairs respectively, Wilcoxon rank sum tests).

### Long intergene distances do not reduce co-expression

**Figure 2:**
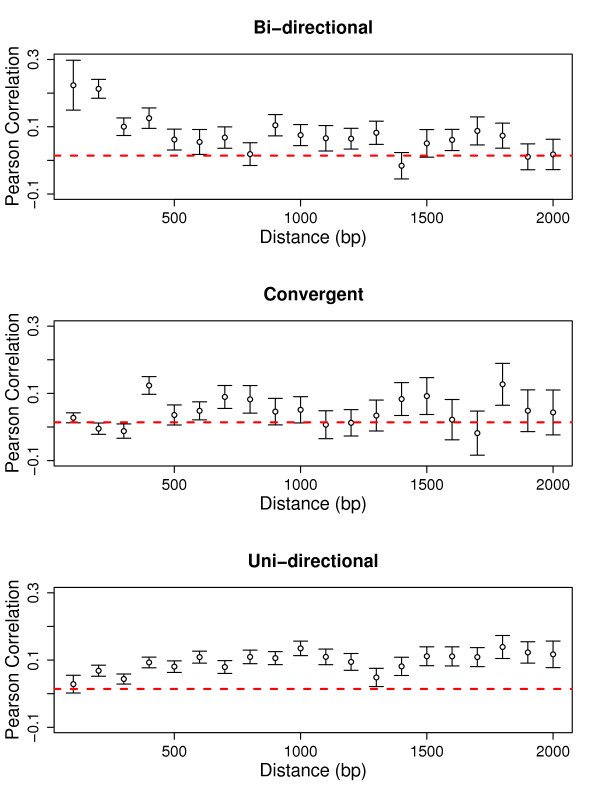
**Distance-dependence of co-expression varies between relative gene orientations**. Dots are means over co-expression of all pairs of direct neighbours in a given distance bin of width 100 bp. Error bars are standard errors of the mean. Dashed lines indicate average co-expression of random gene pairs.

What is the role of shared chromatin domains in promoting the co-expression of neighbouring gene pairs? We can shed some light on this question by examining the distance dependence of co-expression. As we are concerned with transcription initiation and elongation, we defined gene distances as the number of base pairs between the respective transcript boundaries (transcription start and end sites). We did not find any significant correlation between distance and co-expression for neighbouring gene pairs more than 400 bp and less than 2 kb apart (the maximum range for which we had sufficient numbers of gene pairs) (Figure 2; see also [16]). Thus, if chromatin domains do play a role in promoting co-expression, their length must be large or flexible enough to encompass even distant gene neighbours. That co-expression decreases rapidly as soon as two genes are separated by intervening genes (Figure 1) indicates that either chromatin domains usually contain at most two genes, or that more direct interactions between the transcription processes of the neighbours are responsible for co-expression.

The average expression correlation of convergent gene pairs with short intergenic distances was statistically indistinguishable from random gene pairs (Table 2, *P *= 0.50). However, at longer distances (>400 bp), convergent gene pairs did show significant co-expression (as did the other two orientations, *P *= 7.09 × 10^-4^, 2.06 × 10^-7 ^and <2.2 × 10^-16 ^for convergent, bi- and uni- directional gene pairs comparing to random pairs, respectively). For these more distant neighbours, there was actually no difference in the co-expression of convergent and bi-directional pairs (*P *= 0.53). Uni-directional pairs showed stronger co-expression than the other two types at these larger distances (*P *= 5.1 × 10^-4 ^and 2.6 × 10^-4 ^comparing to bi-directional and convergent pairs respectively). This strong co-expression may be more accidental (e.g., transcriptional read-through after failed termination) than functional, as uni-directional pairs are those least conserved in plant evolution [24].

**Table 2:** Co-expression of gene pairs with short and long intergenic distances

	Mean co-expression *	
	
Type	Intergenic distance < 400 bp	Intergenic distance > 400 bp
Bi-directional	0.152	0.060
Uni-directional	0.062	0.100
Convergent	0.024	0.050

* Pearson Correlation; mean co-expression of random gene pairs is 0.014

Direct interactions of the polymerases transcribing two neighbouring genes will be very different for convergent and divergent pairs. However, at distances >400 bp we find no difference between the co-expression of convergently and divergently transcribed gene pairs. This observation is strong evidence that not direct interactions, but shared chromatin regions are in fact responsible for co-expression at these larger distances. Thus, we conclude that co-expression of distant genes (>400 bp) is mostly due to chromatin effects. The cutoff 400 bp was chosen here based on Figure 2; a lower threshold of 300 bp leads to qualitatively very similar results.

### Short bi-directional gene pairs are strongly co-expressed

As seen from Figure 2, the three orientations exhibit very different co-expression patterns at short distances (<400 bp). Bi-directional gene pairs closer than 400 bp are more co-expressed than either more distant bi-directional pairs (*P *= 1.3 × 10^-7^) or close neighbours in other orientations (*P *= 6.8 × 10^-12 ^and *P *= 3.1 × 10^-5 ^for convergent and uni-directional gene pairs, respectively). This is consistent with the existence of shared *cis*-regulatory motifs or promoters in some overlapping promoter regions. At least in yeast [25,26] and mammals [27,28], approximately half of all promoters initiate transcription in both directions, and it is conceivable that the same applies in Arabidopsis.

### Transcriptional interference between short uni-directional and convergent gene pairs

As seen in Figure 2, short convergent as well as short uni-directional gene pairs show reduced co-expression levels compared to more distant pairs of the same orientation (*P *= 0.044 and *P *= 0.0012, respectively). This reduced co-expression suggests some kind of transcriptional interference between the neighbouring genes.

What could be responsible? That this reduced co-expression is found only when at least one of the two genes flankes the intergenic region with its 3' end suggests an involvement of transcription termination. Indeed, transcription by RNA-polymerase II [20] (and probably also polymerase I [18]) usually extends beyond the poly(A) site. In the 'torpedo' model of transcription termination [19], the mRNA is released by cleavage while RNA is still being synthesised. The remaining RNA trails out of the active polymerase, and is chewed up by an exonuclease. Once the 'exonuclease-torpedo' catches up, it dislodges the polymerase, thereby terminating transcription. Thus, the polymerase of an upstream gene may transcribe into the downstream neighbour, interfering with the neighbour's transcription (*e.g.*, by blocking the processing of transcription termination, or by direct collision with another polymerase transcribing the downstream gene).

We further tested the transcriptional interference model by examining overlapping gene pairs on opposing strands. We grouped these into three distinct types according to the TAIR 7 annotation of the *A. thaliana *genome (Table 3). Consistent with the presence of transcriptional interference, mean co-expression of gene pairs with overlaps in their 3' regions (groups 1 and 3) was negative. For group 1, co-expression was significantly lower than that of random gene pairs; for group 3 the difference did not reach statistical significance, possibly due to low sample size (*N *= 17). However, genes overlapping exclusively in their 5' regions (group 2) showed positive co-expression (significantly above group 1, *P *= 0.031, and group 3, *P *= 0.015). Mean co-expression in group 2 was almost as high as for close non-overlapping bi-directional pairs (Table 2). Thus, transcriptional interference seems indeed restricted to the 3' ends of genes, suggesting a role of transcription termination; conversely, the restriction to 3' ends seems not consistent with the simple collision of two polymerases as the source of reduced co-expression.

**Table 3:** Overlapping gene pairs on opposing strands

Group		*N*	Co-expression^a^	mRNA abundance^b^	*P *(compared to random pairs)^c^
**1**	3' overlap	948	-0.019	8.39	0.0020
**2**	5' overlap	23	0.140	7.99	0.142
**3**	fully contained^d^	17	-0.084	7.30	0.103

^a^Average Pearson correlation coefficient

^b ^average mRNA abundance for all genes is 7.73 (log_2 _signal intensity).

^c ^To derive a Null expectation, we analyzed 100,000 pairs of randomly chosen genes; mean Pearson correlation of this random dataset is 0.014. *P*-values from Wilcoxon rank sum tests.

^d ^the shorter gene is fully contained in the longer gene (but transcribed from the opposite DNA strand)

Overlapping gene pairs on different strands (groups 1-3) actually form sense-antisense (SA) pairs. Thus, the lower co-expression of these genes could potentially be explained by RNA interference, which would reduce both the expression abundance and the co-expression of SA pairs. As shown in Table 3, partially overlapping genes are in fact more abundant (group 1) or equally abundant (group 2) as randomly picked genes. This indicates that at least for partially overlapping genes, expression is not strongly influenced by RNA interference (This result remained when we compared overlapping genes to subsets of the full genome with similar gene length, exon number or GO functional category distributions; results not shown).

## Conclusions

Direct neighbours are more often co-expressed than are more distant genes. Beyond approximately 400 bp, the level of co-expression is independent of the intergenic distance, and is the same for divergent and convergent pairs. These results suggest that beyond 400 bp, co-expression is due to shared chromatin domains rather than to direct transcriptional interactions. This chromatin-mediated effect leads to an increase in the expression correlation coefficient of about 0.04 across our set of 1,436 microarray experiments (averaging over the co-expression of convergent and divergent pairs >400 bp apart, and subtracting the co-expression expected for random pairs; uni-directional pairs were excluded here as they are potentially affected by transcriptional read-through even at larger distances).

Using the strength of this effect as a reference, we can get an approximate estimate of the contributions of chromatin-mediated effects and direct interactions. We estimate that for close bi-directional pairs, shared promoter regions are on average responsible for roughly 70% of the co-expression signal ((0.15 - 0.04)/0.15), while the shared chromatin environment accounts for the remaining 30%. Further, for close convergent gene pairs, the co-expression mediated by a shared chromatin environment is reduced by roughly 40% through transcriptional interference ((0.040 - 0.024)/0.040).

As long as there are no intervening genes, chromatin-mediated co-expression does not seem to diminish with increasing intergenic distance. This observation suggests that chromatin domain establishment is regulated such that domains extend as far as needed, regardless of intergenic distance. This conclusion appears consistent with recent results on the role of nucleosome organization and 3-D chromatin structure in gene regulation [29].

At short distances, direct transcriptional interactions become important. For divergent gene pairs, the most important direct interactions are probably the sharing of transcriptional regulatory sites, and bi-directional initiation of RNA-polymerase II transcription from a shared promoter; such bi-directional promoters appear to be the rule rather than an exception in yeast and mammals [30]. That we found elevated co-expression of bi-directional gene pairs only below ~400 bp strongly supports this notion, as this range is similar to the distance found between the two divergent peaks of transcription in other eukaryotes [30]. Beyond promoting co-expression, bi-directional transcription from a single promoter may also serve as a positive feedback loop to support continued expression.

A rather different direct transcriptional interaction is likely responsible for the reduced co-expression found for those pairs where the intergenic region is flanked by at least one 3' gene end (uni-directional and convergent pairs). In transcription termination, the polymerase overshoots the poly(A)-site before being dislodged by a following exonuclease [19,20]; this may result in continued transcription through the intergenic region and into the neighbouring gene, interfering with the neighbour's transcription.

In contrast to previous results in humans and yeast [4,7], we found that uni-directional gene pairs were on average more co-expressed than bi-directional pairs. This is presumably due to the more compact spacing of *A. thaliana *genes, with a higher fraction of closely-spaced uni-directional gene pairs. When comparing the co-expression of the different orientations at fixed intergenic distances, our results agree with those in other eukaryotes.

## Methods

We used release 7 of the *A. thaliana *genome annotation from TAIR [21,22]. Neighbouring gene pairs were defined as protein-coding genes that are direct chromosomal neighbours, with no intervening genes (coding or non-coding). We restricted our dataset to genes that do not overlap with other genes. We also compiled a separate set of overlapping protein-coding gene pairs on opposing strands. We identified putative tandem duplicate genes based on the protocol developed in Ref. [2]: performing pairwise blast searches between all neighbours, we removed those pairs with *E*-value < 0.2 from further analysis. The conservative cutoff 0.2 has been shown previously to lead to good sensitivity and specificity in the identification of even ancient gene duplicates [2].

We downloaded pre-processed expression data from the TAIR database ftp://ftp.arabidopsis.org/home/tair/Microarrays/analyzed_data/affy_data_1436_10132005.zip. The dataset contains 1,436 hybridization experiments using the Affymetrix *A. thaliana *ATH1 (25K) array, which contains 22,810 probe sets. The data were previously normalized using robust multi-array average (RMA) method, according to ftp://ftp.arabidopsis.org/home/tair/Microarrays/analyzed_data/README. Pre-calculated Log 2 values of signal densities were used to calculate co-expression values (Pearson correlation coefficients across all experiments) and expression abundances.

We considered all gene pairs on a given chromosome, i.e., each gene was paired with every other gene on the chromosome. If either of the two genes overlapped with another transcript, the pair was removed from the analysis. Distances were defined (i) as the number of intervening annotated transcripts, or (ii) as the number of base pairs between the transcripts (i.e., between the respective start and end points of transcription).

Co-expression values for individual pairs of genes were defined as Pearson's correlation coefficient between the two expression vectors across normalised hybridisation experiments [1,2,5-7,12-14,16]. To assess general trends of co-expression, we averaged co-expression values over all gene pairs at a given distance (and sometimes with a given orientation). Averaging over signed correlation coefficients does not allow to distinguish lack of co-expression from a superposition of equal amounts of positive and negative co-expression; however, this is justified as the overall distribution of co-expression values is approximately Gaussian (data not shown).

To compare our results to expectations, we constructed a data set of 100,000 pairs of randomly chosen protein-coding genes, for which we also calculated co-expression values. Mean co-expression of this dataset was 0.014. This positive 'random' co-expression is possibly due to the fact that several large gene sets are functionally correlated (such as 'growth-related' or 'stress-related' genes), and hence 'random' pairs usually contain pairs that are positively correlated (while the sets are not necessarily negatively correlated between them).

The statistical significance of differences in mean co-expression values was assessed using Wilcoxon rank sum tests. Throughout the manuscript, we use a threshold α = 0.05 to determine statistical significance. When multiple comparsions were performed (e.g., among the three orientations), we sometimes summarize results by reporting only *P *< the largest *P*-value.

## Authors' contributions

WHC assembled the dataset and carried out the analyses. MJL drafted the manuscript. All authors conceived of and designed the study together through iterative discussions, and read and approved the final manuscript.
